# Whole-brain mapping of long-range inputs to the VIP-expressing inhibitory neurons in the primary motor cortex

**DOI:** 10.3389/fncir.2023.1093066

**Published:** 2023-05-19

**Authors:** Candice Lee, Sandrine L. Côté, Nima Raman, Hritvic Chaudhary, Bryan C. Mercado, Simon X. Chen

**Affiliations:** ^1^Department of Cellular and Molecular Medicine, University of Ottawa, Ottawa, ON, Canada; ^2^Brain and Mind Research Institute, University of Ottawa, Ottawa, ON, Canada; ^3^Center for Neural Dynamics, University of Ottawa, Ottawa, ON, Canada

**Keywords:** monosynaptic circuit tracing, inhibitory neurons, motor cortex, associative learning, orbital frontal cortex (ORB)

## Abstract

The primary motor cortex (MOp) is an important site for motor skill learning. Interestingly, neurons in MOp possess reward-related activity, presumably to facilitate reward-based motor learning. While pyramidal neurons (PNs) and different subtypes of GABAergic inhibitory interneurons (INs) in MOp all undergo cell-type specific plastic changes during motor learning, the vasoactive intestinal peptide-expressing inhibitory interneurons (VIP-INs) in MOp have been shown to preferentially respond to reward and play a critical role in the early phases of motor learning by triggering local circuit plasticity. To understand how VIP-INs might integrate various streams of information, such as sensory, pre-motor, and reward-related inputs, to regulate local plasticity in MOp, we performed monosynaptic rabies tracing experiments and employed an automated cell counting pipeline to generate a comprehensive map of brain-wide inputs to VIP-INs in MOp. We then compared this input profile to the brain-wide inputs to somatostatin-expressing inhibitory interneurons (SST-INs) and parvalbumin-expressing inhibitory interneurons (PV-INs) in MOp. We found that while all cell types received major inputs from sensory, motor, and prefrontal cortical regions, as well as from various thalamic nuclei, VIP-INs received more inputs from the orbital frontal cortex (ORB) – a region associated with reinforcement learning and value predictions. Our findings provide insight on how the brain leverages microcircuit motifs by both integrating and partitioning different streams of long-range input to modulate local circuit activity and plasticity.

## Introduction

The primary motor cortex (MOp) has a well-established role in the execution of voluntary movement (Guo et al., 2015). Recent studies have also identified it as a critical site for motor learning (Xu et al., 2009; Peters et al., 2014; Chen et al., 2015; Kawai et al., 2015). Like other cortical areas, MOp is primarily composed of glutamatergic pyramidal neurons (PNs) and different subtypes of GABAergic inhibitory interneurons (INs), which together form distinctive patterns of local connectivity. In particular, parvalbumin-expressing INs (PV-INs) primarily inhibit the perisomatic region of PNs, somatostatin-expressing INs (SST-INs) primarily inhibit the apical dendrites of PNs, while vasoactive intestinal peptide-expressing interneurons (VIP-INs) mainly inhibit SST-INs and thereby disinhibit PNs. While there are preferential connections between these cell types, the microcircuit connectivity is not entirely unique and specific among cell types (Pfeffer et al., 2013; Pi et al., 2013; Tremblay et al., 2016; Staiger and Petersen, 2021).

During motor learning, both PNs and INs in MOp undergo structural and functional plastic changes (Xu et al., 2009; Peters et al., 2014; Chen et al., 2015; Ren et al., 2022, Yang et al., 2022). Recently, a selective disinhibitory mechanism acting through VIP-IN mediated inhibition of SST-INs has been suggested to promote motor learning by enhancing PN excitability (Adler et al., 2019; Ren et al., 2022). In line with this idea, Ren et al. (2022) have shown that VIP-INs are highly active in the early phase of motor learning while SST-INs show weak activation. Inactivation of VIP-INs in MOp during this early phase impairs learning, demonstrating the importance of VIP-INs for the acquisition of new motor skills (Ren et al., 2022). Interestingly, VIP-INs in MOp have also been shown to represent reward and undergo plastic changes following reward-based associative learning in a non-motor related task. When compared to PNs, PV-INs, and SST-INs, VIP-INs preferentially respond to reward and become more reliably responsive to reward during the associative learning process (Lee et al., 2022). While the plastic changes attributed to VIP-INs in MOp underscore their importance for motor learning, it remains unclear where motor- and reward-related signals to VIP-INs arise from.

Primary motor cortex is heavily interconnected with many cortical and sub-cortical regions (Mao et al., 2011; Hooks et al., 2013; Luo et al., 2019; Duan et al., 2020; Okoro et al., 2022), several of which have been shown to be plastic during motor learning, including the secondary motor cortex (MOs; Cao et al., 2015), anterior lateral motor area (ALM; Chabrol et al., 2019), retrosplenial cortex (RSP; Makino et al., 2017), and thalamus (Biane et al., 2016; Tanaka et al., 2018). In addition, many input regions that project to MOp have also been shown to undergo plastic changes after reward-based associative learning such as the primary somatosensory cortex (Chen et al., 2015), auditory cortex (AUD) (Kisley and Gerstein, 2001; Lee and Rothschild, 2021), RSP (Hattori et al., 2019), ALM, and MOs (Komiyama et al., 2010). Importantly, thus far, these input regions have not been shown to provide preferential input to a specific neuron subtype in MOp.

Here, we utilized a monosynaptic rabies tracing strategy and performed brain-wide mapping of long-range inputs to the four major cell types in MOp (VIP-INs, PV-INs, SST-INs, and PNs). By systematically comparing the proportion of inputs from different brain regions to VIP-INs with the inputs to PV-INs and SST-INs, we found that VIP-INs received significantly more inputs from the orbital frontal cortex (ORB). Considering that both ORB and VIP-INs have been shown to respond to reward (Baltz et al., 2018; Namboodiri et al., 2019; Zhou et al., 2019; Wang et al., 2020; Lee et al., 2022), our results point toward ORB serving as an important node in a reward-related input stream projecting to VIP-INs in MOp. In contrast, SST-INs received more input from the RSP, demonstrating that different IN subtypes receive preferential long-range input from specific brain regions. Taken together, our comprehensive whole-brain mapping uncovers input from ORB that could be responsible for activating VIP-INs in MOp in response to reward and may thereby gate local circuit plasticity during reward-based motor learning.

## Results

**FIGURE 1:**
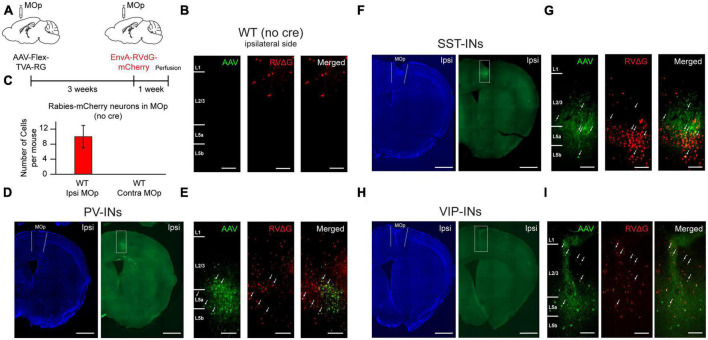
Helper AAV and RV system for retrograde tracing of monosynaptic inputs to PV-INs, SST-INs, and VIP-INs in MOp. **(A)** Helper virus was injected unilaterally into right MOp (ipsilateral) followed by pseudotyped G-deleted rabies virus 3 weeks later. Animals were sacrificed 1 week after the rabies viral injection. **(B)** Example images from a control wild-type mouse injected with AAV2/DJ-hSyn-FLEX-TVA-P2A-eGFP-2A-oG and EnvA-G-deleted-Rabies-mCherry (University of Berlin Viral Core Facility) into ipsilateral MOp. No GFP^+^ labeled cells from the helper virus (left), a small amount of mCherry^+^ labeled cells from the rabies virus (middle), and no co-localized cells (right). The presence of mCherry^+^ cells indicates small TVA leakage. Scale bar, 100 μm. **(C)** Mean number of mCherry^+^ cells in ipsilateral and contralateral MOp of control animals (*n* = 5 mice, 3 sections per mouse). Representative images of the injection sites for PV-Cre **(D,E)**, SST-Cre **(F,G)**, and VIP-Cre **(H,I)** mice injected with AAV2/DJ-hSyn-FLEX-TVA-P2A-eGFP-2A-oG and EnvA-G-deleted-Rabies-mCherry (University of Berlin Viral Core Facility) into ipsilateral MOp. Neurotrace Blue staining (**D,F,H**, left) and GFP fluorescence (**D,F,H**, right) show confinement of GFP^+^ cells to MOp. Zoomed in images **(E,G,I)** from the injection site (dashed rectangle in GFP fluorescent images in panels **D,F,H**) shows GFP^+^ cells (left), mCherry^+^ cells (middle), and colocalized GFP^+^ and mCherry^+^ starter cells (right). Arrows show example starter cells. Scale bars, 1 mm for **(D,F,H)** and 100 μm for **(E,G,I)**.

To identify long-range input regions that are specific to VIP-INs, PV-INs, and SST-INs in the caudal forelimb area of MOp, we utilized the monosynaptic rabies virus (RV) tracing system (Wickersham et al., 2007; Callaway and Luo, 2015; Wall et al., 2016). Helper virus (AAV1-EF1a-DIO-TVA950-T2A-CVS11G or AAV2/DJ-hSyn-FLEX-TVA-P2A-eGFP-2A-oG) was first injected into the right MOp forelimb area of VIP-Cre, PV-Cre, or SST-Cre mice to express avian TVA receptors, rabies glycoprotein (G) and GFP in each cell type, respectively. Three weeks after the helper virus injection, we injected pseudotyped G-deleted RV (EnvA-RVdG-mCherry) into the same site (Figure 1A). One week after the injection of the pseudotyped G-deleted RV, animals were perfused, and coronal sections were imaged at a 120 μm increment across the entire brain (see section “Materials and methods”). It has been shown that the helper virus could have potential leak expression of TVA and subsequent rabies infection in the absence of Cre (Seidler et al., 2008; Callaway and Luo, 2015; Hafner et al., 2019); hence, we conducted control experiments by injecting the helper virus (AAV2/DJ-hSyn-FLEX-TVA-P2A-eGFP-2A-oG) as well as the pseudotyped G-deleted RV (RVdG-EnvA-mCherry, University of Berlin Viral Core Facility) into wild-type (WT) mice and examined whether there were GFP-expressing (GFP^+^) and/or mCherry expressing (mCherry^+^) cells in MOp. We found 0 GFP^+^ cells and an average of 10 mCherry^+^ cells in MOp (Figures 1B, C). When we only injected pseudotyped G-deleted RV in WT mice, we found 0 GFP^+^ and mCherry^+^ cells. Together, these control experiments indicate that there were negligible amounts of TVA leakage at the volume and titer we employed. In contrast to the control experiments, we observed many GFP^+^ cells in PV-, SST-, and VIP-Cre animals, and these GFP^+^ cells were all constrained within the injection site in the MOp. Many of them also co-localized with mCherry, indicating the presence of starter cells (Figures 1D–I). To ensure the specificity of the helper virus to Cre-expressing (Cre^+^) cells, we conducted another set of control experiments, where we injected the helper virus (AAV2/DJ-hSyn-FLEX-TVA-P2A-eGFP-2A-oG) in VIP-Cre:tdTomato mice and examined the fraction of GFP^+^ cells that co-localized with tdTomato. We found that 99% of the GFP^+^ cells co-localized with tdTomato, confirming the specificity of the helper virus to Cre^+^ cells (Supplementary Figures 1A, B).

**FIGURE 2:**
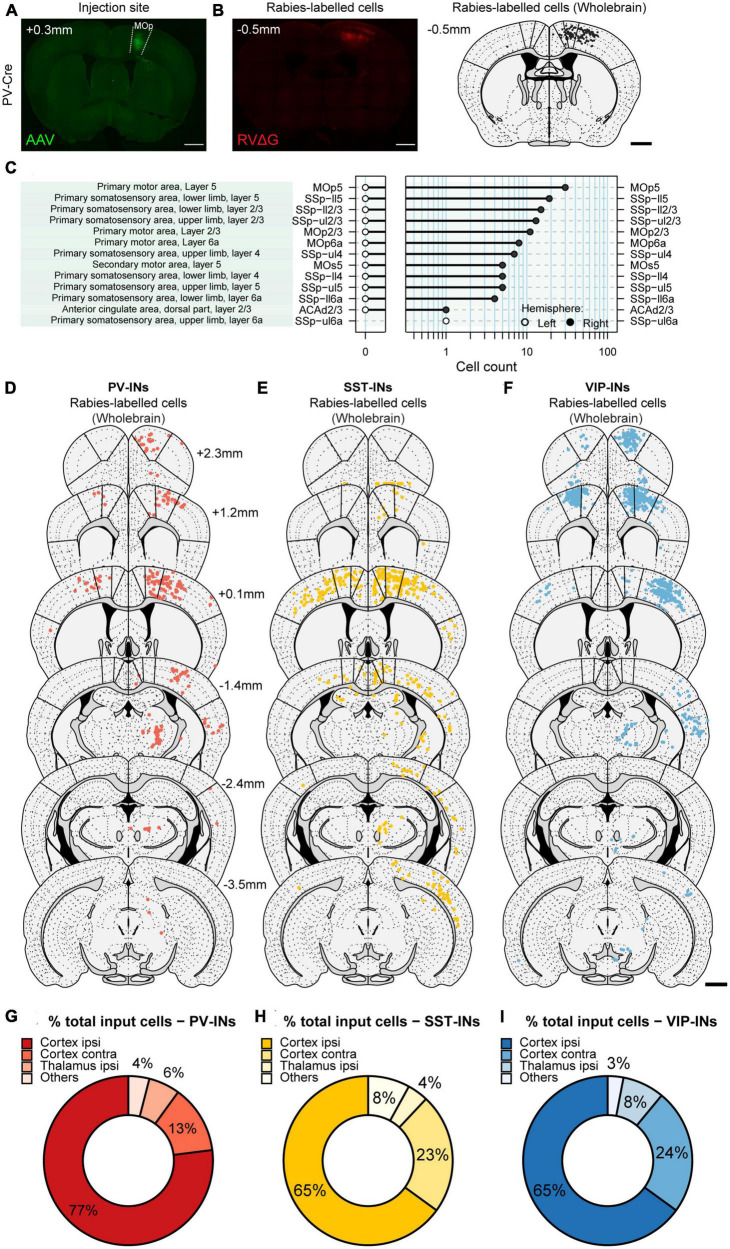
Generating brain-wide input maps to VIP-INs, PV-INs, and SST-INs in MOp. **(A)** Example image of a brain section showing GFP^+^ labeled cells in ipsilateral MOp from the helper virus (AAV2/DJ-hSyn-FLEX-TVA-P2A-eGFP-2A-oG). **(B)** Example image of the same mouse showing mCherry^+^ labeled cells from the rabies virus (EnvA-G-deleted-Rabies-mCherry, University of Berlin Viral Core Facility) (left). Registration of the same section to the Allen Brain Atlas with the Wholebrain software (right). Each black dot is a mCherry^+^ input cell that was automatically detected by the software. **(C)** Cell quantification within different regions, subdivisions, and layers for the example section in panel **(B)**. Example sections showing detected input cells projecting to PV-INs **(D)**, SST-INs **(E)**, and VIP-INs **(F)**. Bold lines indicate the delineation between MOs, MOp, and SSp. Scale bar, 1 mm. Mean percentage of brain-wide inputs to PV-INs **(G)**, SST-INs **(H)**, and VIP-INs **(I)** from broad subdivisions of the brain. Regions with small proportions were grouped together in others. PV-INs, *n* = 4 mice; SST-INs, *n* = 4 mice; VIP-INs, *n* = 5 mice.

To automatically and unbiasedly quantify RV-labeled cells throughout the brain, we employed the software “Wholebrain” (Fürth et al., 2018), which enables automated detection and quantification of labeled neurons. Most importantly, Wholebrain enables scale-invariant registration of brain sections to the Allen Mouse Brain Atlas (Figures 2A–C), thus providing a method to identify all the brain regions from different brain samples within a standardized framework. By applying the Wholebrain software to sections from 2.945 mm anterior to bregma through to 5.055 mm posterior to bregma (cells were not found outside of these coordinates), we generated comprehensive and comparative maps of whole-brain input to VIP-INs, PV-INs, and SST-INs (VIP-INs: 5 mice, ∼35 slices/mouse; PV-INs: 4 mice, ∼48 brain slices/mouse; SST-INs: 4 mice, ∼40 slices/mouse; Figures 2D–F and Supplementary Table 1). To test the reliability of the Wholebrain software in counting the input cells, we compared it to the manual counts done by an experimenter prior to the adoption of the Wholebrain software and found that both methods produced similar counts (Supplementary Figure 2). We first quantified the number of input cells (mCherry^+^ cells outside of MOp) within each region identified in the Allen Brain Atlas, including subdivisions and cortical layers. We found that the total number of labeled input cells varied between each IN subtype (VIP-INs, 3,887 ± 2,021 cells; PV-INs, 1,593 ± 234 cells; SST-INs, 1,253 ± 344 cells). Since the total number of input cells could be dependent on the number of starter cells that were labeled from the viral injection, we also counted the number of starter cells (colocalized GFP^+^ and mCherry^+^ cells within MOp) and calculated an approximate ratio of starter cells to the total number of input cells in the entire brain. We found that the ratio of starter-to-input cells were very similar between the IN subtypes (VIP-INs, 1:15; PV-INs, 1:17; SST-INs, 1:18; Supplementary Table 1).

### Long-range inputs to different IN subtypes in MOp from the cortex

**FIGURE 3:**
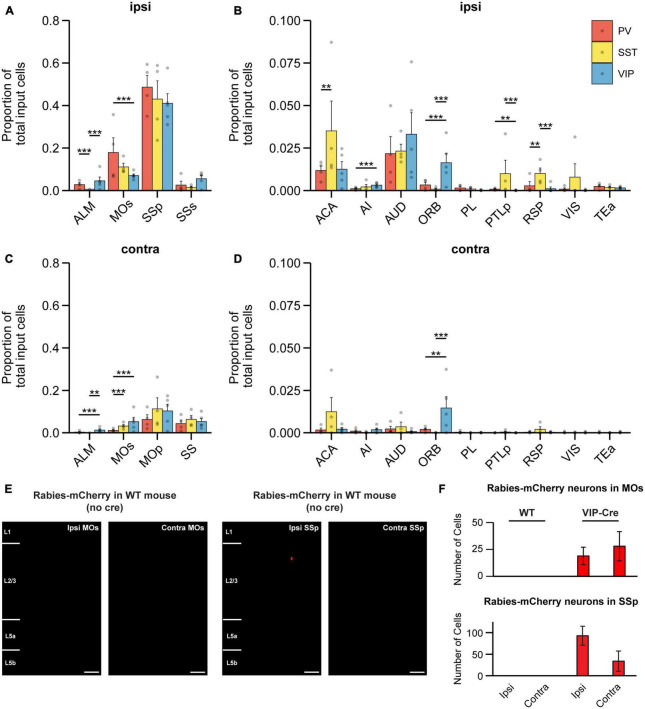
Cortical input to different IN subtypes in MOp. **(A)** Mean proportion of input cells in ipsilateral somatosensory and motor cortices. **(B)** Mean proportion of input cells in other ipsilateral cortical regions. Regions with negligible cell counts are not shown. **(C)** Mean proportion of input cells in contralateral somatosensory and motor cortices. **(D)** Mean proportion of input cells in other contralateral cortical regions. Regions with negligible cell counts are not shown. PV-INs, *n* = 4 mice; SST-INs, *n* = 4 mice; VIP-INs, *n* = 5 mice. Bootstrap with Bonferroni correction for multiple comparisons. Each point represents a mouse. **(E)** Example images of wild-type mice injected with AAV2/DJ-hSyn-FLEX-TVA-P2A-eGFP-2A-oG and EnvA-G-deleted-Rabies-mCherry (University of Berlin Viral Core Facility) into right (ipsilateral) MOp. Almost no mCherry^+^ cells were labeled in MOs and SSp (*n* = 5 mice, 3 sections per mouse). Scale bar, 100 μm. **(F)** mCherry^+^ cells found in MOs and SSp of control and experimental animals (VIP-Cre mice). Error bars show the SEM. **p < 0.01, ***p < 0.001.

We next examined which brain regions each IN subtype receives its input from, beginning with broad subdivisions within the brain. We observed that for all IN subtypes, the majority of input originated in the cortex (Figures 2G–I); in particular, the greatest source of cortical input came from the sensorimotor regions (Figures 3A, C). It is known that ascending tactile sensory information propagates sequentially from the primary somatosensory cortex (SSp) to the secondary somatosensory cortex (SSs). While both regions encode stimulus features, SSp encodes the stimulus more strongly and SSs encodes higher order information such as stimulus-related recall (Condylis et al., 2020) and decision-related activity (Kwon et al., 2016), which is then conveyed back to SSp. Our results show that on the ipsilateral side, the SSp was the largest source of input for all IN subtypes in MOp and comprised 40.99 ± 2.04, 48.64 ± 2.76, and 40.40 ± 4.29% of input to VIP-INs, PV-INs, and SST-INs, respectively (Figure 3A). In contrast, all IN subtypes received substantially less input from SSs (SSp compared to SSs: *p* < 1 × 10^–3^ for all cell types). We also identified major cortical input from the MOs and ALM (also known as frontal MOs). MOs and ALM are two motor regions that show preparatory activity preceding movement and are thought to be akin to the primate premotor cortex (Guo et al., 2015; Li et al., 2015; Chen et al., 2017). We found that MOs provided inputs to all IN subtypes in MOp, and PV-INs received the most among them. Interestingly, we observed that MOs provided more inputs to the INs in MOp than ALM. Specifically, on the ipsilateral side, PV-INs and SST-INs but not VIP-INs received greater input from MOs compared to ALM (VIP-INs, *p* = 0.084; PV-INs and SST-INs, *p* < 1 × 10^–3^; Figure 3A). In contrast, while input from ALM was relatively low compared to MOs, it projected substantially more to PV-INs and VIP-INs than to SST-INs (VIP-IN vs. SST-INs: *p* < 1 × 10^–3^; PV-INs vs. SST-INs: *p* < 1 × 10^–3^; Figure 3A). Hence, unlike SSp and SSs which had similar proportions of input to all IN subtypes in MOp, MOs, and ALM demonstrated some subtype-specific differences. Since MOs and ALM have been shown to have differential roles in movement preparation and execution (Siniscalchi et al., 2016; Chen et al., 2017), our results suggest that specific IN subtypes in MOp may be involved in processing different movement-related information.

Within ipsilateral cortex, we also found other cortical regions that provide differential input biased toward specific IN subtypes in MOp, although they represented a smaller proportion of brain-wide inputs compared to SSp, SSs, MOs, and ALM. The anterior cingulate area (ACA) projected to all IN subtypes but significantly more to SST-INs than to PV-INs, while no significant difference was found between SST-INs and VIP-INs (Figure 3B). The AUD was a major source of input to all three IN subtypes with no significant differences in the proportion of input cells. Intriguingly, the ORB projected significantly more to VIP-INs compared to either PV-INs or SST-INs, and ORB was one of the most pronounced inputs to VIP-INs outside of the SSp (Figure 3B). Lastly, SST-INs received significantly more input from RSP than PV-INs and VIP-INs. In addition, SST-INs also received significantly more inputs from the posterior parietal cortex (PTLp) compared to VIP-INs (Figure 3B). Overall, among ipsilateral cortex, MOs and SSp account for a substantial proportion of brain-wide input to INs in MOp. In addition, we identified biased input from ORB to VIP-INs and RSP to SST-INs.

In the contralateral cortex, MOp was a major source of input to all three IN subtypes (Figure 3C). ALM, MOs, and SS (SSp and SSs combined) had substantially less input cells compared to the ipsilateral side. Unlike ipsilateral ALM which projected similarly to both VIP-INs and PV-INs, contralateral ALM projected more to VIP-INs than to the other two subtypes. Intriguingly, contralateral MOs projected more to VIP-INs and SST-INs than to PV-INs; this is in contrast to ipsilateral MOs, which had a greater proportion of input to PV-INs and less input to SST-INs and VIP-INs. The relative proportion of input from MOs and ALM also differed; unlike the ipsilateral side, where PV-INs and SST-INs but not VIP-INs received more input from MOs compared to ALM, all IN subtypes received more input from contralateral MOs compared to ALM (VIP-INs: *p* = 0.006; SST-INs and PV-INs: *p* < 1 × 10^–3^). These findings demonstrate that subtype-specificity of long-range input from the same region can vary between hemispheres. Outside of ALM, MOs, and SS, the proportion of input from other regions within the contralateral cortex were mostly minimal with some exceptions (Figure 3D). Noticeably, the contralateral ACA trended toward providing more input to SST-INs compared to both PV-INs and VIP-INs, akin to its ipsilateral homolog In addition, contralateral ORB was another exception, as it had a proportion of input cells comparable to its ipsilateral counterpart and also projected significantly more to VIP-INs compared to PV-INs and SST-INs (Figure 3D). Hence, the numerous bilateral inputs from ORB further highlight the importance of this projection to VIP-INs.

To test for the possibility that cre-independent leak expression of TVA could result in non-specific trans-synaptic labeling, we also examined mCherry^+^ cells in the major labeled areas (ipsi and contra MOs and SSp) from our control experiments, in which we injected the helper virus and the pseudotyped G-deleted RV in WT mice (Figure 1B). We observed almost no cells in either region (Figures 3E, F), demonstrating that there was a negligible amount of non-specific trans-synaptic labeling from the TVA leakage at the volume and titer we employed.

### Distinct subregion- and layer-specific inputs to different IN subtypes in MOp

**FIGURE 4:**
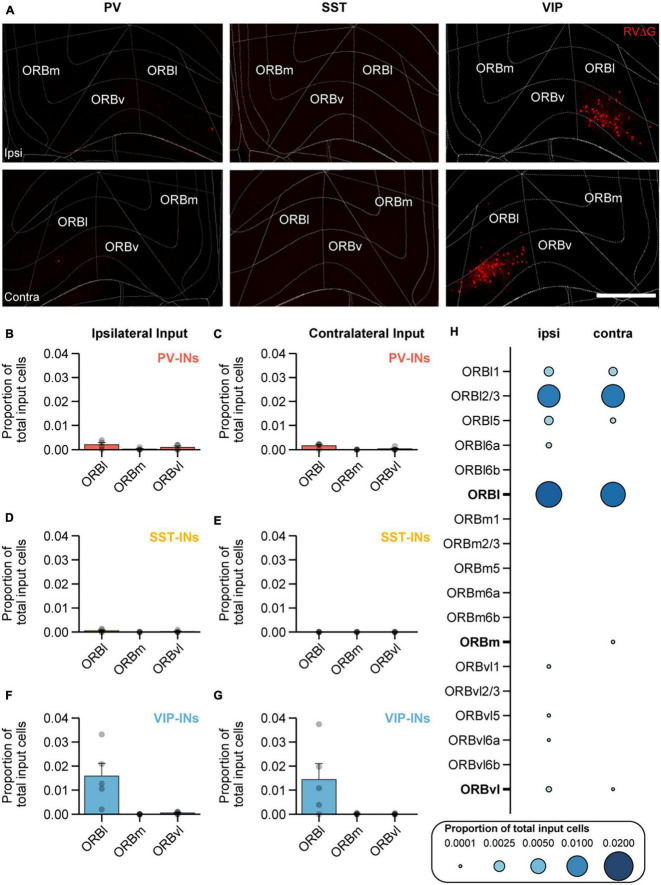
ORBl preferentially projects to VIP-INs in MOp. **(A)** Example images of mCherry^+^ input cells in ORB that project to PV-INs (left), SST-INs (middle),, and VIP-INs (right) in MOp. Top row, ipsilateral ORB. Bottom row, contralateral ORB. Scale bar, 500 μm. Mean proportion of brain-wide inputs from ipsilateral and contralateral ORB to PV-INs **(B,C)**, SST-INs **(D,E)**, and VIP-INs **(F,G)**. **(H)** Mean proportion of brain-wide inputs to VIP-INs found within different layers of ORBl, ORBm,, and ORBvl. Bolded labels show the sum across all layers for each subdivision. PV-INs, *n* = 4 mice; SST-INs, *n* = 4 mice; VIP-INs, *n* = 5 mice. Each point represents a mouse. Error bars show the SEM.

Orbital frontal cortex has been shown to encode and predict value and reward (Baltz et al., 2018; Namboodiri et al., 2019; Zhou et al., 2019; Wang et al., 2020), and previous work has also demonstrated that VIP-INs in MOp undergo plastic changes in response to reward after associative learning (Lee et al., 2022). To better understand this projection, we sought to further examine the location of the input cells within the ORB (Figure 4A). ORB is comprised of three main subdivisions – the lateral, medial, and ventrolateral regions (ORBl, ORBm, and ORBvl). While the function of individual subregions is still unclear, in monkeys, ORBl has been implicated in reward-guided learning, and ORBm has been implicated in reward-guided decision making (Noonan et al., 2010). In rats, ORBvl has been shown to be involved in goal-directed behavior following contingency switches (Parkes et al., 2018; Zimmermann et al., 2018). In our results, we found that on the ipsilateral side, VIP-INs received almost all of its input from ORBl with no detectable input from ORBm and minimal input from ORBvl. The proportion of input cells from ORBl to VIP-INs was significantly higher than to PV-INs and SST-INs (VIP-IN vs. PV-IN: *p* < 0.002; VIP-IN vs. SST-INs: *p* < 1 × 10^−3^; Figures 4B, D, F). Similar observations were made on the contralateral side; VIP-INs also received more input from cells in the contralateral ORBl and barely any input from ORBm and ORBvl. The proportion of input cells from the contralateral ORBl to VIP-INs was also significantly higher than to PV-INs and SST-INs (VIP-IN vs. PV-IN: *p* = 0.007; VIP-IN vs. SST-INs: *p* < 1 × 10^−3^; Figures 4C, E, G). These results indicate that VIP-INs in MOp receive a considerable amount of input from both ipsilateral and contralateral ORBl but not ORBm and ORBvl, consistent with the hypothesis that VIP-INs may be involved in reward-guided motor learning (Figures 4F, G). Since VIP-INs received the most input from ORB, we next asked which layers of ORB project to VIP-INs. We found that on both the ipsilateral and contralateral sides, most of the VIP-IN projecting cells were located in L2/3 of ORBl (Figure 4H). On the ipsilateral side, VIP-INs also received some input from L1, L5, and L6a of ORBl, whereas on the contralateral side, VIP-INs received similar proportions of input from L1 compared to the ipsilateral side but not as much from L5 and L6a (Figure 4H).

**FIGURE 5:**
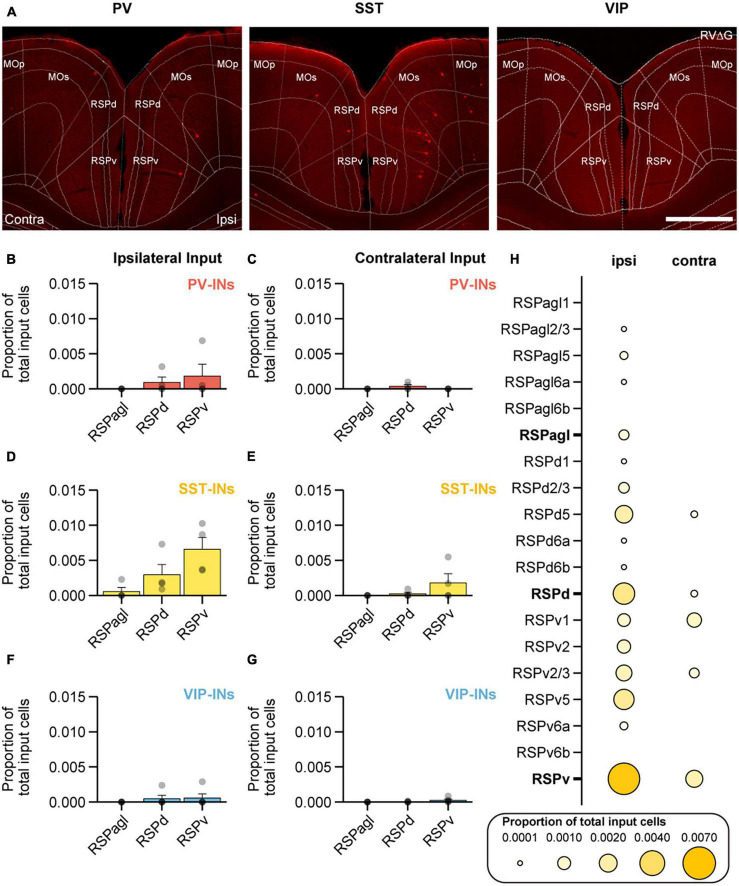
RSPd and RSPv preferentially project to SST-INs in MOp. **(A)** Example images of mCherry^+^ input cells in RSP that project to PV-INs (left), SST-INs (middle), and VIP-INs (right) in MOp. Scale bar, 500 μm. Mean proportion of brain-wide input from ipsilateral and contralateral RSP to PV-INs **(B,C)**, SST-INs **(D,E)**, and VIP-INs **(F,G)**. **(H)** Mean proportion of brain-wide inputs to SST-INs found within different layers of RSPagl, RSPd, and RSPv. Bolded labels show the sum across all layers for each subdivision. PV-INs, *n* = 4 mice; SST-INs, *n* = 4 mice; VIP-INs, *n* = 5 mice. Each point represents a mouse. Each point represents a mouse. Error bars show the SEM.

In addition to ORB, RSP was another region that was identified to be unique as it provided more input to SST-INs compared to both PV-INs and VIP-INs (on the ipsilateral side, SST-INs vs. VIP-INs: *p* < 1 × 10^−3^; SST-INs vs. PV-INs: *p* = 0.015; Figure 3B). RSP is a complex brain region that has been implicated in spatial navigation (Vann et al., 2009), associative learning (Lukoyanov and Lukoyanova, 2006; Makino and Komiyama, 2015; Makino et al., 2017; Hattori et al., 2019; Hattori and Komiyama, 2022), and motor learning (Makino et al., 2017). RSP can be further subdivided into the lateral agranular, dorsal, and ventral parts (RSPagl, RSPd, and RSPv; Figure 5A). Therefore, we also sought to further examine the location of the input cells to SST-INs within the RSP. On the ipsilateral side, SST-INs received the most input from RSPv, followed by RSPd and small amounts of input from RSPagl (Figure 5D). In contrast, PV-INs and VIP-INs received no input from RSPagl and minimal inputs from RSPd and RSPv (Figures 5B, F). On the contralateral side, SST-INs mainly received input from RSPv and almost no input from RSPd and RSPagl (Figure 5E) while PV-INs and VIP-INs received minimal or no inputs from all subdivisions (Figures 5C, G). Given the biased input to SST-INs from RSP, we then sought to determine the laminar distribution of RSP projection neurons to SST-INs (Figure 5H). We observed that in RSPv, input cells were found in all layers on the ipsilateral side except in L6b. Interestingly, on the contralateral side, input neurons were only found in L1 and L2/3.

### Long-range inputs to different IN subtypes in MOp from the thalamus

**FIGURE 6:**
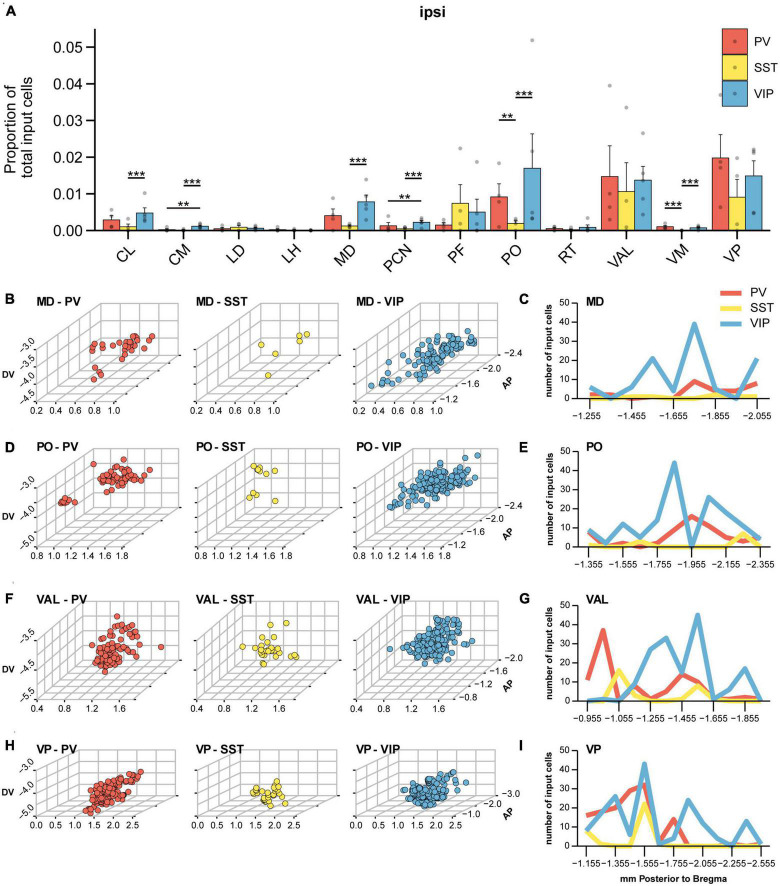
Thalamic input to IN subtypes in MOp. **(A)** Mean proportion of brain-wide input found in different thalamic nuclei. Nuclei with a negligible number of cells are not shown. Bootstrap with Bonferroni correction for multiple comparisons. Each point represents a mouse. **(B)** Three-dimensional spatial distribution of input cells within MD of PV-INs (left), SST-INs (middle), and VIP-INs (right). Axes correspond to the medial-lateral (ML), dorsal-ventral (DV), and anterior-posterior (AP) coordinates relative to bregma. Each point represents a cell. **(C)** Number of cells within MD along the anterior-posterior axis for different IN subtypes. **(D)** Three-dimensional spatial distribution of input cells within PO of PV-INs (left), SST-INs (middle), and VIP-INs (right). **(E)** Number of cells within PO along the anterior-posterior axis. **(F)** Three-dimensional spatial distribution of input cells within VAL of PV-INs (left), SST-INs (middle), and VIP-INs (right). **(G)** Number of cells within VAL along the anterior-posterior axis. **(H)** Three-dimensional spatial distribution of input cells within VP of PV-INs (left), SST-INs (middle), and VIP-INs (right). **(I)** Number of cells within VP along the anterior-posterior axis. PV-INs, *n* = 4 mice; SST-INs, *n* = 4 mice; VIP-INs, *n* = 5 mice. Error bars show the SEM. **p < 0.01, ***p < 0.001.

While the majority of brain-wide input to all three IN subtypes originated from cortex, ipsilateral thalamus was the next largest source of input to VIP-INs, PV-INs, and SST-INs and provided 8, 6, and 4% of the total inputs to VIP-INs, PV-INs, and SST-INs, respectively (Figures 2G–I). In contrast, we did not find many input cells in the contralateral thalamus. Inputs from various thalamic nuclei to MOp have been shown before, including significant input from motor-related nuclei such as ventral anterior lateral (VAL) and ventromedial (VM) nuclei (Hooks et al., 2013; Lam and Sherman, 2015; Duan et al., 2020; Muñoz-Castañeda et al., 2021), as well as some input from sensory thalamic nuclei such as the ventral posterior (VP) nuclei (Hunnicutt et al., 2014; Muñoz-Castañeda et al., 2021) and posterior (PO) nuclei (Hooks et al., 2013) among others. From all the nuclei in the thalamus, we observed that a greater proportion of inputs arose from the mediodorsal (MD), parafascicular (PF), PO, VAL, and VP nuclei of thalamus, while relatively small amounts of inputs arose from the central lateral (CL), central medial (CM), lateral dorsal (LD), lateral habenula (LH), paracentral nucleus (PCN), reticular nucleus (RT), and ventromedial (VM) nuclei of thalamus (Figure 6A). Intriguingly, we also observed some subtype specificity in the proportion of input. For example, CL projected more to VIP-INs than to SST-INs, and CM provided modest but significantly more input to VIP-INs than to either PV-INs or SST-INs. MD projected significantly more to VIP-INs compared to SST-INs but not to PV-INs, and PCN also provided modest input that was biased to VIP-INs. PO and VM projected significantly more to VIP-INs and PV-INs compared to SST-INs. Lastly, LD, LH, PF, RT, VAL, and VP did not show any subtype specific biases.

It has been shown that various nuclei within thalamus are topographically organized (Angaut et al., 1985; Pierret et al., 2000; Veinante et al., 2000; Lam and Sherman, 2015); hence, we mapped the input neurons within each of the major input nuclei in three-dimensional space to assess whether there is spatial specificity or clustering among the input neurons. Previous work has shown that MD neurons projecting to PV-INs and VIP-INs in the prefrontal cortex occupy distinct locations along the medial-lateral axis (Mukherjee et al., 2021). Here, we found that MD neurons projecting to PV-INs and VIP-INs in MOp were intermingled along the medial-lateral axis (Figure 6B). However, MD neurons projecting to VIP-INs appeared to be more widely distributed along the anterior-posterior axis while neurons projecting to PV-INs were located more toward the posterior end of the nucleus (Figures 6B, C). Previous work has shown that first-order POm (the medial division of PO) receives direct input from the brainstem and is located more anteriorly (centered around ∼1.7 mm posterior to bregma). Higher-order POm, on the other hand, does not receive input from the brainstem and is located more posteriorly (centered on 2.2 mm posterior to bregma; El-Boustani et al., 2020). We found that input neurons to VIP-INs were located within both of these subdivisions, while neurons providing input to PV-INs lay in between these two subdivisions (Figures 6D, E). The ventral group of thalamic nuclei including VAL, VM, and VP show primarily sensory- and motor-related activity. In addition, within the ventral-lateral subdivision, the anterior regions strongly represent whisking-related activity, and more posterior regions are more limb-related (Tlamsa and Brumberg, 2010). We found that VAL was among the most largest source of input to all three IN subtypes, and a cluster of PV-IN projecting neurons was found in the most anterior parts of VAL. In contrast, VIP-IN projecting neurons were found to be more distributed throughout the middle and most posterior regions of VAL (Figures 6F, G). Noticeably, SST-INs received a smaller proportion of input from MD, PO, and VAL (Figure 6A). The posterior division of the ventral group (VP) relays sensory-related input to the somatosensory cortex (Jensen and Killackey, 1987; Lee and Sherman, 2008). VP neurons projecting to PV-INs were located in the most anterior parts through to the center of the nucleus along the anterior-posterior axis. VIP-IN-projecting neurons in VP were found more uniformly throughout the anterior-posterior length of VP, and SST-IN-projecting neurons were sparser and located just anterior to the center of the nucleus (Figures 6H, I). Interestingly, the center region (∼−1.555 mm posterior to bregma) appears to have the largest concentration of input neurons, regardless of the IN subtype. Overall, these results demonstrate that INs in M1 receive input from both first and higher order thalamic nuclei, as well as from sensory, motor, and polymodal nuclei. Moreover, neurons in thalamus projecting to different IN cell types in MOp can occupy distinct regions within the same nucleus.

### Brain-wide long-range inputs to PNs in MOp

**FIGURE 7:**
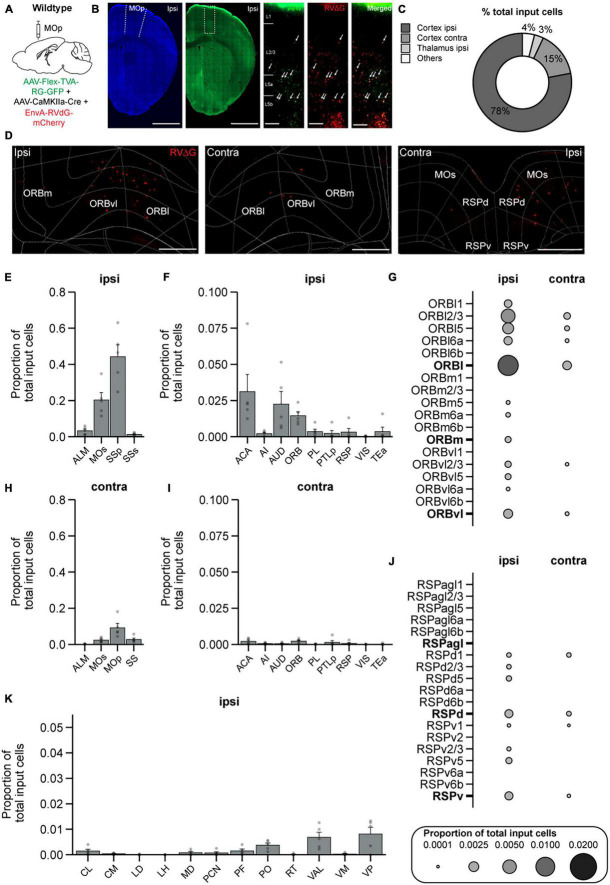
Brain-wide input to PNs in MOp. **(A)** Helper virus (AAV2/DJ-hSyn-FLEX-TVA-P2A-eGFP-2A-oG) and CaMKII-Cre were injected unilaterally into right (ipsilateral) MOp of wild-type mice followed by EnvA-G-deleted-Rabies-mCherry (University of Berlin Viral Core Facility) 3 weeks later. Animals were sacrificed 1 week after the rabies viral injection. **(B)** Representative images of the injection site. Neurotrace Blue staining and GFP fluorescence show confinement of GFP^+^ cells to MOp. Zoomed in view from the injection site (dashed rectangle in GFP fluorescent image) shows GFP^+^ cells (left), mCherry^+^ cells (middle), and colocalized GFP^+^ and mCherry^+^ starter cells (right). Arrows show example starter cells. Scale bars = 1mm and 100 μm. **(C)** Mean percentage of brain-wide inputs to PNs from broad subdivisions of the brain. **(D)** Example images of mCherry^+^ input cells in ipsilateral ORB (left), contralateral ORB (middle), and bilateral RSP that project to PNs (right). Scale bars, 500 μm **(E)** Mean proportion of brain-wide input cells in ipsilateral somatosensory and motor cortices. **(F)** Mean proportion of input cells in other ipsilateral cortical regions. Regions with negligible cell counts are not shown. **(G)** Mean proportion of input cells in contralateral somatosensory and motor cortices. **(H)** Mean proportion of input cells in other contralateral cortical regions. Regions with negligible cell counts are not included. **(I)** Mean proportion of brain-wide inputs to PNs found within different layers of ORBl, ORBm, and ORBvl. Bolded labels show the sum across all layers of each subdivision. **(J)** Mean proportion of brain-wide inputs to PNs found within different layers of RSPagl, RSPd, and RSPv. Bolded labels show the sum across all layers of each subdivision. **(K)** Mean proportion of brain-wide input to PNs found in different thalamic nuclei. *n* = 5 mice. Each point represents a mouse. Error bars show the SEM.

Lastly, we performed a similar experiment and generated a brain-wide map of input to PNs to examine if any regions send exclusive inputs to IN subtypes but not to PNs. We injected AAV-CaMKII-Cre and the AAV helper virus (AAV2/DJ-hSyn-FLEX-TVA-P2A-eGFP-2A-oG) in WT mice, and 3 weeks after the helper virus injection, we injected the pseudotyped G-deleted RV (EnvA-RVdG-mCherry) into the same site (Figure 7A). Starter cells were constrained to MOp (Figure 7B). We again used the Wholebrain software to unbiasedly count the labeled input cells in all brain regions (Figures 7C, D). We found a starter-to-input cell ratio of 1:9, and the total number of input cells for PNs was 3,431 ± 1,671 cells (PNs: 5 mice, ∼47 slices/mouse). We found that PNs in MOp received very similar inputs compared to all IN subtypes, which includes a large proportion of input from the ipsilateral SSp and MOs (Figures 7E, H) and a smaller proportion from the ACA and AUD (Figures 7F, I). PNs also received a considerable amount of input from ORB as previously shown (Hooks et al., 2013); however in contrast to VIP-INs, ORB input to PNs was primarily ipsilateral, and input cells were located throughout all the layers in ORBl except for L6b (Figure 7G). In addition, similar to SST-INs, PNs also received input from RSP but it was proportionally distributed across RSPd and RSPv (Figure 7J). Lastly, many of the major thalamic input nuclei to INs in MOp also projected to PNs, including PO, VAL, and VP, which is consistent with the literature (Hooks et al., 2013; Hunnicutt et al., 2014; Tanaka et al., 2018; Figure 7K). It has been shown that CaMKII can be detected in INs (Veres et al., 2023); therefore, we also performed control experiments to examine if we also see AAV helper virus expression in INs. We injected AAV-CaMKII-Cre and the AAV helper virus (AAV2/DJ-hSyn-FLEX-TVA-P2A-eGFP-2A-oG) in WT mice and then stained for GABA to identify INs. We found that ∼14% of the GFP-expressing cells were GABA positive (Supplementary Figures 1C, D). Since the ratio of starter cells (GFP^+^/mCherry^+^) to TVA-expressing cells (GFP^+^) in the PN brain samples is 1:3, our results suggest that only a tiny proportion of the starter cells (∼4.7%) will be INs in this experiment. Overall, from our tracing experiments, we observed that the majority of the input regions to PNs were similar to IN subtypes.

## Discussion

It is well-established that MOp is involved in the execution of volitional movement; however, recent observations that MOp also exhibits reward-related activity that could facilitate reward-based motor learning have only begun to be explored. Previous work using *in vivo* two-photon imaging to compare reward representations among PNs, VIP-INs, PV-INs, and SST-INs, revealed that VIP-INs in MOp are preferentially responsive to reward compared to the other cell types in MOp, and their responses to reward become more reliable after associative learning (Lee et al., 2022). Hence, in this study, we employed monosynaptic rabies tracing and brain-wide mapping to identify candidate brain regions with preferential projections to VIP-INs in MOp, which might confer reward-related input to VIP-INs. By generating a comprehensive and subtype-specific map of brain-wide inputs to VIP-INs and comparing it to the input maps for PV-INs and SST-INs, we demonstrated that the major inputs to all three IN subtypes originated from sensory, motor, prefrontal cortices, as well as thalamus. While many of the identified brain regions provided input to all three IN cell types, some regions were significantly biased toward one particular IN cell type. Among these, we observed dense bilateral input from ORB projecting primarily to VIP-INs. In comparison, we also found biased input from the ipsilateral RSP to SST-INs. Through the generation of IN subtype-specific brain-wide input maps to MOp, this study provides a framework for future investigations exploring how different IN subtypes in MOp integrate long-range inputs from various brain regions and consequently, influence motor output and motor learning.

While the use of monosynaptic rabies tracing provides a number of unique advantages, there are several important technical considerations. It has previously been shown that TVA – EnvA interactions are extremely efficient; hence, very small levels of cre-independent leak expression of TVA can be sufficient for viral infection by EnvA-pseudotyped virus (Seidler et al., 2008; Callaway and Luo, 2015; Hafner et al., 2019). For example, Hafner et al. (2019) conducted a series of control experiments where they injected cre-dependent and flp-dependent helper virus to express TVA but not rabies glycoprotein in WT mice followed by EnvA-RVdG injection. The authors found that there were still cells in the injection site labeled by RVdG virus, demonstrating that small amounts of TVA “invisible leak” expression can enable RVdG infection, and these cells could be mistakenly deemed to be local pre-synaptic inputs. However, cells with cre-independent leak of TVA did not show any trans-synaptic labeling. We also conducted control experiments by injecting the helper virus and RVdG in WT animals. While we did observe some mCherry-only expressing cells in MOp, we did not observe any trans-synaptic spread resulting from TVA-leakage (Figures 3E, F). To avoid potential confounds, we refrained from quantifying mCherry-only expressing cells in ipsilateral MOp in our analyses.

It is also important to mention that the transfection efficiency can vary significantly as the result of numerous factors (promoter, viral titer, injection protocols, injection efficacy, the cell type targeted, and transgenic mouse lines used); therefore, the number of starter cells can vary between different studies as well as between animals within a single study (Callaway and Luo, 2015; Roelofs et al., 2021). For this reason, it is convention to view the results as ratios instead of absolute numbers. We found our ratio of starter:input cells comparable to other studies that examined presynaptic input to interneurons in cortical regions (Wall et al., 2016; Gehrlach et al., 2020). In other studies that did not report the starter:input cell ratio, we found that we had a similar number of starter cells per section (Duan et al., 2020). Moreover, Yao et al. (2023) found that differences in the number of starter cells do not drastically alter the proportions of inputs from pre-synaptic input regions, likely due to cells receiving convergent input. With these technical caveats and considerations in mind, we discuss the conceptual implications of our findings below.

One major finding from our work is that ORB provides dense bilateral input preferentially to VIP-INs, and ipsilateral input to PNs. Although previous work identified ORB input to PNs in the vibrissal region of MOp, it remained unclear whether ORB inputs also synapse onto other cell types. Using fluorescent retrograde microbeads, Hooks et al. (2013) found that ipsilateral ORB input neurons projecting to MOp largely originated in deep layers of ORB, while contralateral input was predominantly from superficial layers of ORB. In addition, anterograde tracing of ORBl and ORBvl projections demonstrated the presence of ORB axons throughout all layers of ipsilateral MOp with the greatest presence of axons in deep L5B and L6 (Hooks et al., 2013). In the present study, we found that PNs and VIP-INs are the major recipient cell types of ORB input to MOp, and that both receive input mainly from the lateral part of ORB (ORBl). Interestingly, ORBl neurons projecting to PNs were more numerous on the ipsilateral side and were more evenly distributed throughout L1 to L6a. In contrast, ORB input neurons projecting to VIP-INs were unique in that they resided mainly in the L2/3 of both ipsilateral and contralateral ORBl. These results suggest that the anatomical differences in laminar and hemispheric connectivity from ORB to PNs and VIP-INs in MOp could be related to different roles in processing reward-related signals during learning.

The exact functional role of ORB is still unclear but it is thought to be involved in predicting or updating expected outcomes or values during learning (Baltz et al., 2018; Zhou et al., 2019). Neurons in ORB have been shown to persistently represent both cue and reward throughout learning in an auditory-cued reward associative learning task, and photoinhibition of ORB specifically during the reward-predicting cue period impaired behavioral performance during learning (Namboodiri et al., 2019). Another study found that during an odor-cued reward associative learning task, 29% of ORB neurons demonstrated large amplitude responses to the cue odor after associative learning (Wang et al., 2020). Interestingly, if naïve mice were exposed to the same odor prior to learning and in the absence of reward, only 11% of neurons in ORB showed a response, and these responses were lower in amplitude and trial-to-trial consistency compared to after learning. In line with the notion that ORB represents value, the authors found that ORB neuron responses to the cue were greater when the mice were thirsty compared to when they were satiated. Furthermore, optogenetic silencing of ORB during the cue and response period reduced cue-evoked anticipatory licking, suggesting ORB is involved in encoding the relative value of a stimulus and choosing an appropriate response (Wang et al., 2020). Together with our anatomical results, these findings signify that neurons in ORB represent value by integrating internal state with learned associations and might be involved in reward-based motor skill learning.

It has also been hypothesized that ORB may have a role in regulating reinforcement learning through top-down modulation of cortex (Banerjee et al., 2020; Liu et al., 2020). However, the different subdivisions of ORB are infrequently investigated in isolation, and their individual functions remain unclear. One study found that in monkeys, ORBl is necessary for reward-based learning while ORBm is necessary for reward-based decision making (Noonan et al., 2010). In SSp, ORBl has been shown to project strongly to L2/3 and L5 (Banerjee et al., 2020). Similar to the cue and reward-related responses we observed in MOp during an auditory-cued classical conditioning task (Lee et al., 2022), a subpopulation of neurons in SSp showed outcome or reward-related activity during a texture-based go/no-go task. Furthermore, these outcome-related responses in SSp underwent ORB-dependent remapping during a reversal learning task, indicating that ORBl input to SSp may function as a teaching signal to modulate and remap SSp activity during reward-based reversal learning. In line with their findings, our results suggest that during motor learning, a bilateral teaching signal from ORBl to VIP-INs in MOp could mediate broad disinhibition. Recent findings from Ren et al. (2022) and Szadai et al. (2022) show that VIP-INs are key regulators in gating a cortex-wide response to reward. Therefore, input from ORB to VIP-INs in MOp during cue and reward could be a potential mechanism for driving VIP-INs and gating plastic changes in MOp during reward-based motor learning.

Generally, other studies mapping presynaptic inputs to different GABAergic inhibitory interneurons in cortex have not found biased input to specific cell types (Wall et al., 2016; but see Leinweber et al., 2017; Ährlund-Richter et al., 2019; Duan et al., 2020; Yao et al., 2023). One possibility is that there are region-specific differences in long-range connectivity. A previous study also used rabies tracing to map presynaptic inputs to VIP-INs, PV-INs, and SST-INs in MOp; however, the results are discordant with some of our key findings. In Duan et al. (2020), they did not observe preferential inputs from ORB to VIP-INs in MOp, but instead, they found that ORB projected mostly to PV-INs, followed by VIP-INs, and then SST-INs. The authors also found that ipsilateral input from ORB to VIP-INs was substantially more abundant than contralateral input, while we found ipsilateral and contralateral inputs from ORB to VIP-INs were comparable in proportion (Figures 4F, G). Additionally, Duan et al. found that ORBvl was the primary source of ORB input to MOp, followed by ORBl input, and little to no input from ORBm. Incongruously, we found that ORB input to MOp arose mainly from ORBl with very small amounts of input from ORBvl and ORBm (Figures 4F, G). After carefully examining the experimental procedures, we observed several differences in methods including a significant difference in the injection coordinates. In our study, we targeted MOp using the coordinates 0.3 mm anterior and 1.5 mm lateral to bregma. This coordinate was determined based on previous motor mapping studies (defined by measuring evoked forelimb movement) using either optogenetic (Harrison et al., 2012) or electrical (Tennant et al., 2011; Brown et al., 2022) stimulation. This coordinate evoked forelimb movement most reliably (Harrison et al., 2012) and is near the center of the forelimb motor representations (Tennant et al., 2011; Brown et al., 2022). Furthermore, using *in vivo* two-photon imaging, several studies have observed motor learning-related plasticity during forelimb motor learning tasks using this coordinate (Peters et al., 2014, 2017; Chen et al., 2015; Yang et al., 2022), and optogenetic inhibition at this coordinate impairs forelimb function (Peters et al., 2014). Lastly, we also previously observed reward responses in VIP-INs and associative learning-related plasticity in different neuronal subtypes at this coordinate (Lee et al., 2022), which supports our observations that input from ORB to VIP-INs in MOp could provide reward-related signals during reward-based motor learning. In contrast to our injection site, Duan et al. used the coordinate of 1.34 mm anterior and 1.75 mm lateral to bregma. While stimulation at this coordinate can evoke forelimb movement, it is located at the edge of the forelimb representation (Tennant et al., 2011; Harrison et al., 2012; Brown et al., 2022) and is more strongly associated with jaw movement (Tennant et al., 2011). Additionally, Duan et al. used a dorsal-ventral coordinate of −1.5 mm which would target L6. While INs can be found throughout all layers of cortex, VIP-INs, SST-INs, and PV-INs are most numerous in L2–5 (Kawaguchi and Kubota, 1996; Kawaguchi, 1997; Prönneke et al., 2015; Tremblay et al., 2016); therefore, we performed our injections at two depths of −0.3 and −0.5 mm, targeting both L2/3 and L5 of MOp, respectively. Hence, the differential observations from the two studies regarding the cell-type specificity of post-synaptic targets in MOp and bilateral projection patterns may further highlight how different IN subtypes are uniquely involved in regulating local circuitry in different layers and regions of MOp. Finally, we noticed that the two studies utilized different viruses from different sources. Duan et al. used two helper viruses, one to express TVA receptors and another to express RG whereas we utilized a single helper virus to express both TVA and RG concurrently; hence, different serotypes of AAV could also possibly introduce unforeseen biases.

In this study, we have identified a multitude of inputs that target different cell types within MOp. The brain-wide maps of inputs revealed long-range connectivity onto different IN subtypes and provide insight on how input from different regions is parsed within the MOp microcircuitry. Importantly, we identify ORB as a putative candidate region that could drive the reward representation among VIP-INs within MOp. This suggests that MOp is not only involved in producing motor commands, but also integrates numerous streams of complex input, including sensory and reinforcement-related information, to modulate motor behavior and motor learning. Many inputs from a single brain region project onto several IN subtypes; therefore, these streams could engage different cell types in MOp based on the behavioral state or context. Future work will involve detailed investigation of the functional connectivity of these long-range inputs during behavior to examine whether and how they engage distinct modules within MOp during associative and motor learning.

## Materials and methods

### Mouse lines

All animal experiments were approved by the University of Ottawa Animal Care Committee and in accordance with the Canadian Council on Animal Care guidelines. Experimental mice were group-housed in plastic cages with food and water *ad libitum* in a room with a reversed light cycle (12–12 h). PV-Cre (JAX 008069), SST-Cre (JAX 013044), VIP-Cre (JAX 010908), Ai14 (JAX 007914), and B6129SF1/J (JAX 101043) mouse lines were acquired from Jackson Laboratory (Bar Harbor, ME, USA). VIP-Cre:Ai14 mouse colonies were generated by crossing VIP-Cre females with Ai14 males. All Cre mouse lines were homozygous and in C57BL/6 × 129S4 background, and both male and female mice were used.

### Surgery

Mice underwent two surgeries. In the first surgery, they were injected with a helper virus. After 3 weeks, in the second surgery, animals were injected with an engineered RV. The same surgical procedures were used for both surgeries on the same injection site. Mice were deeply anesthetized using 1–2% isoflurane and given a subcutaneous injection of buprenorphine (0.05 mg/kg) for analgesia. An incision was made, and a small craniotomy was performed at the coordinate 1.5 mm lateral and 0.3 mm anterior to bregma above the forelimb area of MOp. A glass pipette was loaded and lowered to 500 μm below the pia and 100 nl of the virus was injected at a rate of 10 nl/min. The pipette was left in place for 10 min to avoid backflow, then the pipette was raised to 300 μm below the pia and an additional 100 nl of the virus was injected. The pipette was again left in place for 10 min. All injections were performed on the right hemisphere only. The incision was then sutured, bupivacaine ointment was applied topically, and mice recovered on a heated pad. Four hours following surgery, an additional subcutaneous injection of buprenorphine (0.05 mg/kg) was given. For the helper virus injections in PV-Cre, SST-Cre, and VIP-Cre, either AAV1-EF1a-DIO-TVA950-T2A-CVS11G (plasmid obtained from Friedrich Miescher Institute for Biomedical Research Vector Core, titer 3.11 × 10^13^ GC/ml) or AAV2/DJ-hSyn-FLEX-TVA-P2A-eGFP-2A-oG (Canadian Neurophotonics Platform Viral Vector Core Facility, titer 1.2 × 10^13^ GC/ml) was used. We switched to a new helper virus as the initial one became unavailable during the span of our experiments. For the helper virus injections targeting PNs, a 1:1 mixture of AAV2/DJ-hSyn-FLEX-TVA-P2A-eGFP-2A-oG and AAV9.CamKII-Cre.SV40 (Addgene, titer 2.1 × 10^13^ GC/ml or UPenn Vector Core, titer 2.8 × 10^12^ GC/ml) was used. For the engineered RV injection, EnvA-G-deleted-Rabies-mCherry (Salk Institute for Biological Studies, titer 3.95 × 10^8^ or 4.24 × 10^7^ GC/ml or University of Berlin Viral Core Facility, titer 2.4 × 10^8^ GC/ml) was used.

### Histology

One week after the rabies injection, mice were deeply anesthetized and transcardial perfusion was performed with 4% paraformaldehyde (PFA). Brains were kept in 4% PFA overnight at 4°C and transferred to a 30% sucrose and 0.1% sodium azide in phosphate buffered saline (PBS) solution at 4°C. The bottom right side of the brain was cut approximately 1–2 mm deep to mark the injected hemisphere. Brains were then sectioned with a microtome with the thickness of 40 μm and kept in 0.1% sodium azide in PBS solution. Every fourth section was mounted such that the entire brain was screened at 120 μm intervals. Sections were then counterstained using either Neurotrace Blue 435/455 Blue Fluorescent Nissl Stain (Thermo Fisher Scientific) or Vectashield Hardset Antifade Mounting Medium with DAPI (Vector Laboratories).

### Control experiments

For all the control experiments, the same surgery, histology and imaging methods were consistent with the experimental groups described above. To test for TVA leakage, AAV2/DJ-hSyn-FLEX-TVA-P2A-eGFP-2A-oG (Canadian Neurophotonics Platform Viral Vector Core Facility) and EnvA-G-deleted-Rabies-mCherry (University of Berlin Viral Core Facility) were injected in WT (B6129SF1/J) mice. After 3 weeks, mice were perfused, and histology, imaging and analyses were performed. To test that helper virus expression was specific to Cre-expressing cells, AAV2/DJ-hSyn-FLEX-TVA-P2A-eGFP-2A-oG (Canadian Neurophotonics Platform Viral Vector Core Facility) was injected in *VIP-Cre:Ail4* mice. After 3 weeks, mice were perfused, and histology, imaging and analyses were performed. To test for RVdG leakage, EnvA-G-deleted-Rabies-mCherry (University of Berlin Viral Core Facility) was injected in WT mice. After 1 week, mice were perfused, and histology, imaging and analyses were performed. To test if the CaMKII promoter is specific to PNs, we injected a 1:1 mixture of AAV2/DJ-hSyn-FLEX-TVA-P2A-eGFP-2A-oG and AAV9.CamKII-Cre.SV40 (Addgene #105558). After 3 weeks, mice were perfused and sectioned. For immunofluorescence targeting GABA, rabbit anti-GABA (1:500; Millipore, A2052) was used for the primary antibody and Alexa Fluor Plus 594 donkey anti-rabbit (1:500; Invitrogen, A21207) was used for the secondary antibody.

### Imaging

Images were obtained at 10× with either the Zeiss AxioImager M2, Zeiss AxioScanner Z1 or Zeiss AxioObserver 7 microscopes. Entire brain sections were tiled using motorized stage controls and stitched using Zeiss ZEN Microscope Software.

### Data analysis

Starter cells were identified as cells with colocalized eGFP, mCherry, and DAPI/Neurotrace Blue and counted manually using the multi-point tool in Fiji (Supplementary Figure 1; Schindelin et al., 2012). Input cells were identified as cells outside of right MOp with colocalized mCherry and DAPI/Neurotrace Blue. Input cells were detected, counted, and registered to brain regions using WholeBrain Software Suite (Fürth et al., 2018) in R. All cell quantifications and image registrations were manually inspected and adjusted as needed. Any incorrectly detected cells that did not colocalize with the counterstain (DAPI or Neurotrace Blue) were removed from the dataset. These incorrectly detected “cells” were mainly autofluorescence signals with fluorescent intensities similar to those of the real cells. Subsequent analysis and figures were made using custom-written code in R and Matlab. All analyses were performed on the proportion of total input cells for each region unless otherwise stated. To calculate the proportion, the number of input cells in a specified region was divided by the total number of input cells in the entire brain for each animal.

### Statistics

Comparisons between IN subtypes were performed using one-sided bootstrap. Briefly, distributions *F* and *G*, were sampled with replacement and compared under the null hypothesis *H*_0_ : *F* = *G* for 1,000 replications. The achieved significance level was calculated as the proportion of replications supporting the null hypothesis (Efron and Tibshirani, 1994). *p*-Values were corrected for multiple comparisons using the Bonferroni correction. All statistics were performed in Matlab.

## Data availability statement

The original contributions presented in this study are included in the article/Supplementary material, further inquiries can be directed to the corresponding author.

## Ethics statement

The animal study was reviewed and approved by the Animal Care and Veterinary Service (ACVS).

## Author contributions

CL, SLC, and SXC conceived the project and wrote the manuscript. CL and SLC conducted the experiments. CL, SLC, NR, HC, and BM performed the image registration and cell quantifications. CL and SLC analyzed the data under the supervision of SXC. All authors contributed to the article and approved the submitted version.

## Supplementary material

Supplementary Figure 1 Control experiments assessing the specificity of the viruses. **(A)** Example images from a VIP-Cre-tdTomato mouse injected with AAV2/DJ-hSyn-FLEX-TVA-P2A-eGFP-2A-oG showing the injection site in right MOp and a zoomed in view with GFP^+^ cells labeled from the helper virus (left), tdTomato^+^ cells (middle), and a merged image showing co-localized GFP^+^ and tdTomato^+^ cells (right). Scale bars, 1 mm and 50 μm. **(B)** Mean percentage of GFP^+^ cells co-localizing with tdTomato (*n* = 4 mice, 2 sections per mouse). **(C)** Example images from a wild-type mouse injected with AAV2/DJ-hSyn-FLEX-TVA-P2A-eGFP-2A-oG and CaMKII-Cre showing the injection site in right MOp and a zoomed in view with of GFP^+^ cells (left), GABA^+^ cells (middle), and a merged image showing co-localized GFP^+^ cells and GABA^+^ cells (right). **(D)** Mean percentage of GFP^+^ cells co-localized with GABA (*n* = 4 mice, 2 sections per mouse).

Supplementary Figure 2 Comparison of manual and Wholebrain software counts. Example whole brain sections from VIP-Cre **(A)**, PV-Cre **(B)**, SST-Cre **(C)**, and B6129SF1/J (PN) **(D)** animals displaying manual counts, automatic counts made by the Wholebrain software and the number of “cells” that were removed manually due to being incorrectly detected by the software.

Supplementary Table 1 Individual counts of mCherry^+^ cells outside of MOp and starter cells for each cell type and their ratio. Summary of the animals, mCherry^+^ cells, starter cells, and number of brain slices with cells that were analyzed for each experimental group in this study.
