# Supplementary figures and images for: Whole-brain mapping of long-range inputs to the VIP-expressing inhibitory neurons in the primary motor cortex
Source: Front Neural Circuits. 2023 May 19;17:1093066. doi: 10.3389/fncir.2023.1093066 (PMC10237295; doi:10.3389/fncir.2023.1093066)

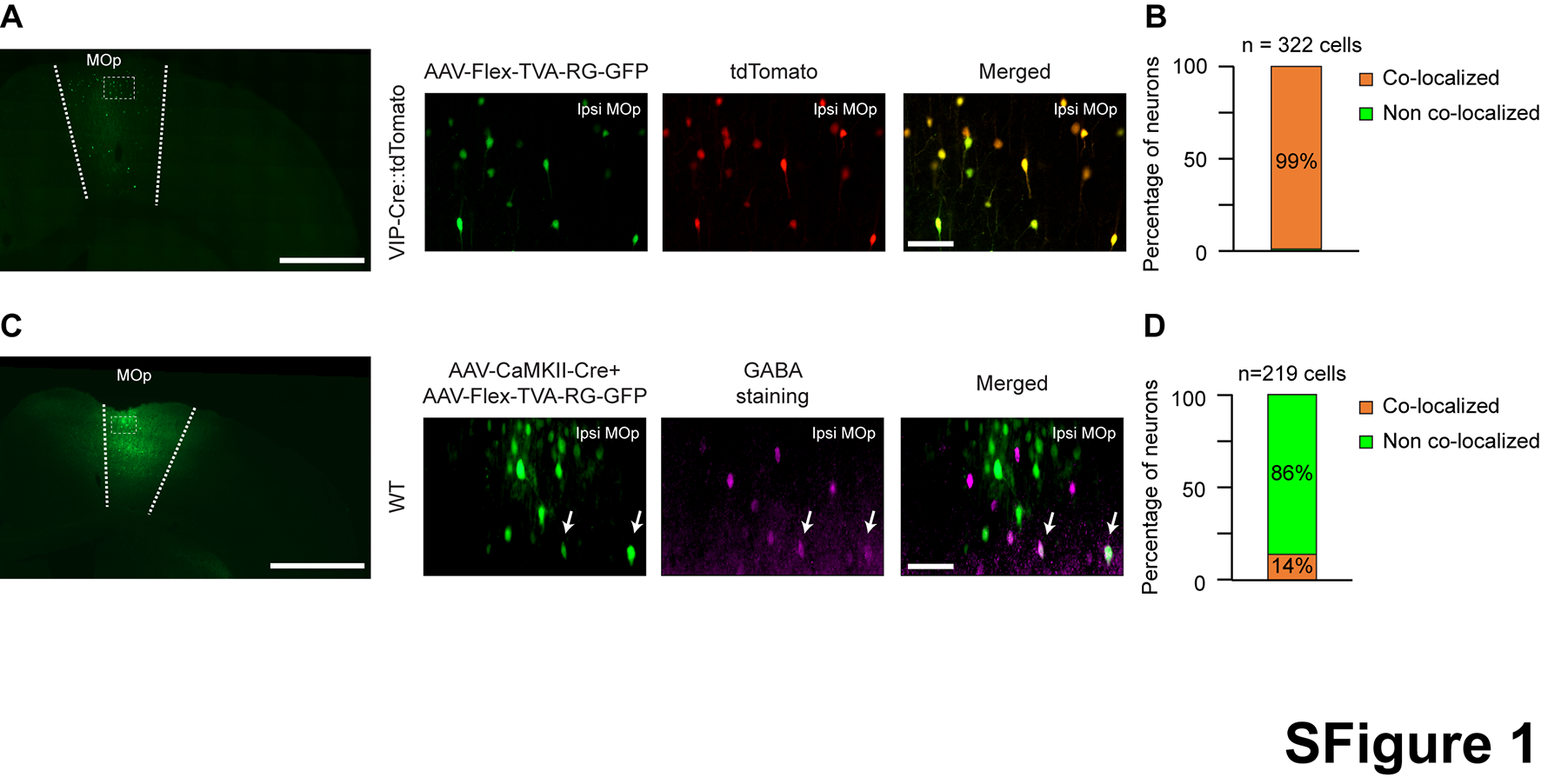

Supplement: Supplementary Figure 1 — Control experiments assessing the specificity of the viruses. (A) Example images from a VIP-Cre-tdTomato mouse injected with AAV2/DJ-hSyn-FLEX-TVA-P2A-eGFP-2A-oG showing the injection site in right MOp and a zoomed in view with GFP+ cells labeled from the helper virus (left), tdTomato+ cells (middle), and a merged image showing co-localized GFP+ and tdTomato+ cells (right). Scale bars, 1 mm and 50 μm. (B) Mean percentage of GFP+ cells co-localizing with tdTomato (n = 4 mice, 2 sections per mouse). (C) Example images from a wild-type mouse injected with AAV2/DJ-hSyn-FLEX-TVA-P2A-eGFP-2A-oG and CaMKII-Cre showing the injection site in right MOp and a zoomed in view with of GFP+ cells (left), GABA+ cells (middle), and a merged image showing co-localized GFP+ cells and GABA+ cells (right). (D) Mean percentage of GFP+ cells co-localized with GABA (n = 4 mice, 2 sections per mouse). [file Image_1.tif]

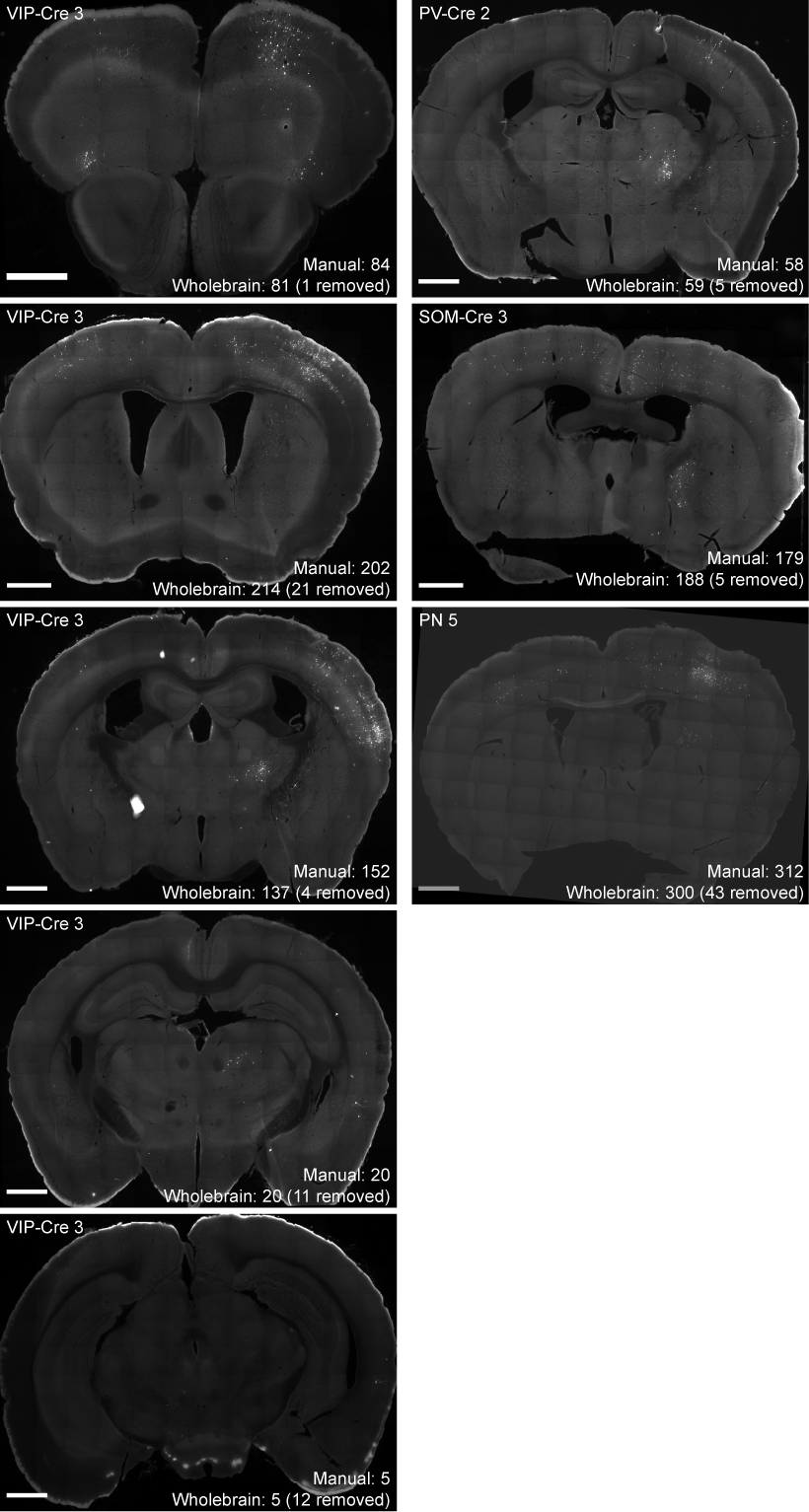

Supplement: Supplementary Figure 2 — Comparison of manual and Wholebrain software counts. Example whole brain sections from VIP-Cre (A), PV-Cre (B), SST-Cre (C), and B6129SF1/J (PN) (D) animals displaying manual counts, automatic counts made by the Wholebrain software and the number of “cells” that were removed manually due to being incorrectly detected by the software. [file Image_2.TIF]
