# Supplementary material for: Whole-brain mapping of long-range inputs to the VIP-expressing inhibitory neurons in the primary motor cortex
Source: Front Neural Circuits. 2023 May 19;17:1093066. doi: 10.3389/fncir.2023.1093066 (PMC10237295; doi:10.3389/fncir.2023.1093066)
Supplement: Supplementary Table 1 — Individual counts of mCherry+ cells outside of MOp and starter cells for each cell type and their ratio. Summary of the animals, mCherry+ cells, starter cells, and number of brain slices with cells that were analyzed for each experimental group in this study. [file Data_Sheet_1.PDF]

Supplementary Table 1. Individual counts of mCherry^{+} cells outside of MOp and starter cells for each cell type and their ratio.

	**VIP-IN**			
Animal number	number of slices with cells analyzed	mCherry^{+} cells	starter cells (GFP^{+}/mCherry^{+})	ratio
1	33	1396	94	1:15
2	39	1798		
3	25	3696		
4	38	11746		
5	40	1018		

	**PV-IN**			
Animal number	number of slices with cells analyzed	mCherry^{+} cells	starter cells (GFP^{+}/mCherry^{+})	ratio
1	44	1418		
2	50	2043		
3	51	1016	66	1:15
4	59	1894	99	1:19

	**SST-IN**			
Animal number	number of slices with cells analyzed	mCherry^{+} cells	starter cells (GFP^{+}/mCherry^{+})	ratio
1	44	1171		
2	39	725	40	1:18
3	37	2190		
4	39	1115		

	**PN**			
Animal number	number of slices with cells analyzed	mCherry^{+} cells	starter cells (GFP^{+}/mCherry^{+})	ratio
1	42	2558	344	1:7
2	41	2291	337	1:7
3	45	1505	292	1:5
4	38	801	263	1:3
5	69	10002	1165	1:9
